# Genetic variability in G2 and F2 region between biological clones of human respiratory syncytial virus with or without host immune selection pressure

**DOI:** 10.1590/0074-02760140299

**Published:** 2015-02

**Authors:** Claudia Trigo Pedroso Moraes, Danielle Bruna Leal Oliveira, Angelica Cristine Almeida Campos, Patricia Alves Bosso, Hildener Nogueira Lima, Klaus Eberhard Stewien, Alfredo Elias Gilio, Sandra Elisabete Vieira, Viviane Fongaro Botosso, Edison Luiz Durigon

**Affiliations:** 1 Instituto de Ciências Biomédicas; 2 Instituto Butantan, São Paulo, SP, Brasil; 3 Hospital Universitário, Universidade de São Paulo, São Paulo, SP, Brasil

**Keywords:** HRSV, genetic variability, immune selection pressure

## Abstract

Human respiratory syncytial virus (HRSV) is an important respiratory pathogens among children between zero-five years old. Host immunity and viral genetic variability are important factors that can make vaccine production difficult. In this work, differences between biological clones of HRSV were detected in clinical samples in the absence and presence of serum collected from children in the convalescent phase of the illness and from their biological mothers. Viral clones were selected by plaque assay in the absence and presence of serum and nucleotide sequences of the G2 and F2 genes of HRSV biological clones were compared. One non-synonymous mutation was found in the F gene (Ile5Asn) in one clone of an HRSV-B sample and one non-synonymous mutation was found in the G gene (Ser291Pro) in four clones of the same HRSV-B sample. Only one of these clones was obtained after treatment with the child's serum. In addition, some synonymous mutations were determined in two clones of the HRSV-A samples. In conclusion, it is possible that minor sequences could be selected by host antibodies contributing to the HRSV evolutionary process, hampering the development of an effective vaccine, since we verify the same codon alteration in absence and presence of human sera in individual clones of BR-85 sample.

Human respiratory syncytial virus (HRSV) is one of the most important respiratory pathogen (Graham 2011) since the infection rate in young children approaches 70% in the first year of life (Tripp 2004). New infections throughout life are common, although disease symptoms are milder in healthy adults (Hall et al. 2001 ). In 2009, the World Health Organization (WHO) estimated that 64 million infants and young children are infected by HRSV annually in the world and that 160,000 of them die every year (WHO 2009 ). There is no commercial vaccine to prevent infection with HRSV (Collins & Melero 2011 , Graham 2011). Important factors should be considered to explain unsuccessful attempts in the development of effective vaccine against HRSV, such as the immaturity of the immune system, the suppressor effect of the mother's antibody in the first years of life (Collins & Graham 2008, Graham 2011) and viral genetic variability (Peret et al. 1998). The most immunogenic proteins are G (adhesion) and F (fusion), which show the highest rates of amino acid variability in their outer portions (Ogra 2004). Both are important candidates in the development of an effective vaccine or prophylactic immunotherapy (Graham 2011). Therefore, some intrapopulation variation can also occur in HRSV samples after passage in cell culture, without immune pressure, changing viral susceptibility to neutralisation by anti-F MAb (Marsh et al. 2007). In addition, differences between the G gene from clones obtained from the same sample of bovine respiratory syncytial virus in the absence of immune pressure have also been detected (Deplanche et al. 2007). Taking into account that G and F contain important epitopes for the development of preventive immunotherapy and vaccines and that the genetic variability of these proteins should be considered for successful protection against HRSV, the aim of this work was to determine the genetic variations of the G2 and F2 regions in biological clones of the same viral population of HRSV obtained from children. In addition, we examined if preexisting mutants are selected by antibodies present in the sera collected from the same children and from their respective biological mothers.

Nasopharyngeal aspirates were obtained from children hospitalised in University Hospital of São Paulo University (HU-USP), in the city of São Paulo, Brazil, during 1998, as previously described (Vieira et al. 2001). Informed consent was obtained from the parents or guardians of the three children enrolled in this study and ethical guidelines for human experimentation were strictly observed. Three HRSV samples (BR98-22, BR98-8, BR98-85) were randomly chosen for this study. In addition, 3 mL of blood were collected from children in the convalescent phase of the HRSV infection and from their biological mothers. All six sera (from each mother and child) analysed by immunofluorescent assay against the isolated virus showed the presence of IgG-specific antibodies to HRSV. No antibodies were detected to HEp-2 and VERO cells showing that all sera were appropriate for the assays.

The titres of the HRSV strains and sera were firstly determined by quantitative plaque assay. Afterwards, plaque forming units obtained in the presence or absence of human serum were picked up and propagated in HEp-2 cells. The G2 region (nt 597- 903) was sequenced in 50 clones and the F2 region (nt 1-595) in 77 clones (Table I). Clones from the Br98-83 sample did not show any alteration in nucleotide sequences of F and G genes, either in the presence or absence of serum. Furthermore, no mutations were observed in clones from Br98-22 in the presence of serum. On the other hand, one synonymous mutation at position 705 (C705T) was observed in the sequences of gene G obtained from clone 22-cl13 of the Br98-22 sample, in the absence of serum, where Ser^230^ was maintained (Table II, Supplementary Fig. 1). Synonymous codon alterations are important because they can affect mRNA stability, rate of translation and post-modifications of proteins (Shabalina et al. 2013). These mutations contributing to virus antigenic preservation, replication and spread of mRNA virus population at host environment (Lauring et al. 2012).

**TABLE I:** Number of human respiratory syncytial virus (HRSV) clones picked up and propagated in HEp-2 cells and number of partial sequences of F and G genes obtained in the absence and presence of serum from mother and child

	Br98-22				Br98-83				Br98-85		
HRSV strains	WS	CS	MS		WS	CS	MS		WS	CS	MS
Total picked clones	41	13	2		81	8	4		46	4	5
Total clones propagated in cell culture	32	8	2		46	5	4		46	4	5
Sequence analysis of G2	6	4	1		16	1	1		18	3	0
Sequence analysis of F2	20	0	0		30	0	0		27	0	0

CS: child's serum; MS: mother's serum; WS: without serum.

**TABLE II:** Nucleotide and amino acid changes in G and F genes of human respiratory syncytial virus (HRSV) clones

	Clone with mutation	Mutation in F gene			Mutation in G gene		
Sample		WS	With MS	With CS	WS	With MS	With CS
Br98-22	22-cl13	-	-	-	C705T	-	-
							
Br98-85	85-cl 11	T14A (Ile5Asn)	-	-	-	-	-
	85-cl 20 85-cl 13	- -	- -	- -	G903A T886C (Ser291Pro)	- -	- -
	85-cl 24	-	-	-	T886C (Ser291Pro)	-	-
	85-CS 3	-	-	-	-	-	T886C (Ser291Pro)

the number in parentheses indicates the amino acids changes. CS: child's serum; MS: mother's serum; WS: without serum.

Four biological clones from Br98-85 exhibited nucleotide alterations in the absence of serum (Table II, Supplementary Figs 2-4). The clone 85 cl-20 showed one transition at position 903 (G903A) of gene G which did not alter the stop codon at position 296 of the G protein. The clone 85-cl11 showed a transversion in gene F (T14A) leading to a change from Ile to Asn at position 5 of the F protein and consequently the addition of a potential site for *N*-glycosylation (0.6952, with jury agreement 9/9 - by NetNGlyc program).

Finally, two clones (85-cl 13 and 85-cl24) showed one non-synonymous modification at position 886 leading to a change at residue 291 from Ser, a potential site for *O*-glycosylation (G-score 0.694; I-score 0.027), to Pro. The same alteration was found in clone 85CS3 in the presence of child's serum (Table II). Ser is the predominant amino acid in most samples sequenced recently in this position, so this change represents a reversion of codon Ser^291^ to Pro^291^, the previous codon, corroborating the "flip-flop" phylogenetic pattern (Botosso et al. 2009). *In silico* analysis using the NetOGlyc program showed that this alteration led to the loss of the potential for *O*-linked oligosaccharides at position 291 of the G protein.

A limitation in this study was the amount of sera that was only enough to carry out one passage. This limitation involving works using human sera has also been reported (Shinoff et al. 2008). Moreover, polyclonal antibodies react with multiple antigenic regions of proteins, which can hamper the detection of the genetic region responsible for the generation of escape mutants (Lambkin et al. 1994, Sullender & Edwards 1999).

The nucleotide differences between some clones and the master sequence either in presence or absence of sera observed in this work (Table II) could indicate quasispecies presence in the viral population, as suggested by other authors after analyses of gene G (Yui et al. 2003) and gene F (Marsh et al. 2007) from a HRSV population in vivo. Besides, mixed sequences of G gene have already found in the same patient (Agoti et al. 2010), indicate that some nucleotides can be replaced in consequence of host immune pressure.

The minority sequences in a viral population can be selected by neutralising antibodies (Ruiz-Jarabo et al. 2000) and they can become dominant in a viral population. Besides, it was demonstrated that a limited number of genetic alterations in both G and F genes are necessary to make the virus resistant to polyclonal antibodies in cell culture (Sullender & Edwards 1999, Tomé et al. 2012). Considering these facts, our results are important since they indicate that virus from the same population can also replicate itself using alternative codons.

Besides, they can be selected by human serum, even if just one amino acid has been altered, showing the adaptability of this virus.

In summary, we detected mutants in the same viral population and determined if human sera collected from children in the convalescent phase and from their biological mothers could select them. Two synonymous mutations were found in the G protein in the absence of serum, one of them in different HRSV-A samples. On the other hand, two non-synonymous alterations (Ser291Pro in G protein and Ile→5Asn in F protein) were found in three clones of HRVS-B in the absence of serum and in one clone in the presence of child's serum. This finding suggests that minority HRSV sequences can be selected by the host immune system over time. Information on intrapopulation HRSV variation could be important in understanding the genetic diversity of this virus. However, more studies are necessary to understand HRSV quasispecies dynamics and the relation of viral mutation with human antibody pressure, since immunotherapy and vaccine success also depend on these factors.
